# The Drug Treatment of Disease

**Published:** 1914-11-14

**Authors:** 


### The Hospital

The Workers' Newspaper of Administrative Medicine and Institutional Life, Administration, National Insurance and Health.

No. 1482, Vol. LVII. SATURDAY, NOVEMBER 14, 1914. PRICE ONE PENNY.

### THE DRUG TREATMENT OF DISEASE.

A curious chapter—on the whole, perhaps, not a very fruitful one—could be written on the history of the basis of scientific therapeutics. The record would display some widely-separated extremes, and the moderate and rational mean, though not altogether absent, would hardly be the most conspicuous feature. In such a recital would be recorded a story of widespread credulity, and of a polypharmacy which embraced many substances of animal origin and almost the entire vegetable series, "from the cedar tree that is in Lebanon even unto the hyssop that springeth out of the wall." This simple-minded faith was followed in due course by a widespread scepticism, which coincided, in general terms, with an increasing attention to pathological anatomy. Practitioners whose student days date back to the eighties and nineties of the last century will readily recall how the wreck and ruin of the *post-mortem* room was used as a conclusive argument against the possibility of modifying such conditions by the administration of drugs. Under such an influence faith in medicinal agents sank, at least in many instances, to the vanishing point, and anything like therapeutic enthusiasm excited little more than a contemptuous smile. The text-books of the day reflected the operation of the same influence, and while describing in elaborate detail the pathological changes found in diseased organs after death, dealt with treatment only in vague and general terms. If clinical observation was not entirely regarded as a department of natural history and a meditation upon methods of dying, it was certainly often pursued without any considerable therapeutic hopefulness. Reaction had in its turn produced reaction, and universal confidence was replaced by a widespread and sceptical fatalism.

While it is possible with a fair amount of accuracy to describe in general terms the therapeutic record of the past, it is less easy to be sure of the history of our own day. We, in our turn, must submit ourselves to the judgment of those who succeed us; and, on the whole, we may be sure of a just product. We think, however, it may be fairly said that we shall hardly be condemned for our extremes; and while time and experience may perhaps prove some of the arguments to be inadequate, we shall not be found wholly divorced from logical processes. If we have escaped the "doctrine of signatures" and other mediæval formulas, we have also, at least in some measure, refused the therapeutic helplessness of our immediate predecessors. This medium position may be largely traced to two influences. First, we have learned that the contemplation of anatomical changes is not the whole story of pathology. These changes show the ruin which has been wrought, but not the nature of the destructive forces. Pathology, in a word, has been driven to its proper duty, and has been recognised as the study not merely of the *post-mortem* facts associated with disease, but also of the processes of which these facts are the final expression. Necessarily, when disease in its early active phases ceases to be regarded as structural change and becomes recognised as disturbed or abnormal activities, the prospect of modifying its course by drugs or other agents takes on a more hopeful hue. A proposal to restore a house in ruins by turning on it a stream of water will excite only a pitying smile, but the same suggestion when the house is burning receives quite a different welcome. In some such fashion pathology, now that it has become a study of processes and causes, has given a more hopeful basis to treatment, just as in its gloomy anatomical days it seemed to impose a hopeless and unrelieved therapeutic sterility.

Another influence which has given encouragement to the use of drugs in the treatment of disease is the pursuit of exact scientific observations both in the laboratory and at the bedside. The unbelief which was at one time widely prevalent never had a sufficient justification, but it could only be fully overcome by forces appropriate to the circumstances of the day. In the scientific atmosphere of the medical curriculum the student became accustomed to demand actual demonstration and measurement, and where these could not be afforded he hesitated to give his faith. To pretend that these conditions have been satisfied in respect to any large number of drugs would be absurd, but it is equally certain that they have been satisfied in some instances; and the influence of such a position has an encouraging effect on drug treatment in general.

Take, for example, the present pharmacological and therapeutic position of the digitalis group, as compared with what was taught, say, ten or fifteen years ago. Instead of vague propositions we have precise and confident knowledge, and are able to judge exactly where these remedies will be appropriate and what will be their effects. Not for a moment do we suggest that individual practitioners had not before more recent days learned the skilful use of these and other remedies. But such knowledge was largely a personal acquisition, and it rested mainly on an empirical basis, whereas to-day the student may be taught well-defined principles, and may have the validity of these demonstrated at the bedside by methods which fulfil the most exact requirements of modern science. This is indeed a change which cannot fail to diffuse a more sanguine atmosphere over the therapeutic area in general.

Laboratory experiments with drugs have perhaps no immediate appeal to the practising physician, but even these must exercise some influence on the therapeutic outlook. An interest of the same order may be found in a lecture on "The Action of Drugs on Plants," recently delivered to the Royal Society of Medicine by Professor J. C. Bose. The lecture throughout is highly interesting and suggestive, and the experiments it describes have a direct bearing on such questions as the apparently contradictory effects produced by different doses of the same drug, idiosyncrasies in drug action, and so on. There is plain evidence that the introduction of a new factor, as a drug or chemical, into the environment of the plant cells may be employed as a means of arousing or diminishing cellular activities, and if this is true of vegetable cells it must apply equally to those of the animal kingdom.

